# Association Between White Matter Hyperintensities and Chronic Kidney Disease: A Systematic Review and Meta-Analysis

**DOI:** 10.3389/fmed.2022.770184

**Published:** 2022-05-03

**Authors:** Cun-Sheng Wei, Cai-Yun Yan, Xiao-Rong Yu, Lin Wang, Rui Zhang, Jun-Ying Jiang, Qi Dai, Jun-Rong Li, Xue Mei Chen

**Affiliations:** ^1^Department of Neurology, The Affiliated Jiangning Hospital With Nanjing Medical University, Nanjing, China; ^2^Department of Oncology, The Affiliated Sir Run Run Hospital of Nanjing Medical University, Nanjing, China

**Keywords:** chronic kidney disease, cerebral small vessel disease, systematic review, white matter hyperintensities, meta-analysis

## Abstract

**Objectives:**

Previous studies of the associations between white matter hyperintensities (WMH) and chronic kidney disease (CKD) were still conflicting; therefore, our study aimed to conduct a systematic review of all of the available research on this topic and a meta-analysis of the association between WMH and CKD among observational studies.

**Setting and Design:**

Systematic review and meta-analysis.

**Outcome Measures:**

Severity of WMH.

**Methods and Participants:**

All relevant studies in public databases were examined until 15 November 2020. Two independent reviewers assessed all the included studies using the Cross-Sectional/Prevalence Study Quality (CSSQ) scale, and then literature review and meta-analyses were undertaken.

**Results:**

We pooled the odds ratio (OR) for the presence of WMH, periventricular hyperintensities (PVH), and deep subcortical white matter hyperintensities (DWMH) of patients with CKD vs. non-CKD patients by subgroup analysis, and the results obtained were WMH OR 2.07, 95% CI [1.58, 2.70], PVH OR 2.41, 95% CI [1.90, 3.05], and DWMH OR 2.11, 95% CI [1.60, 2.80], respectively. The main outcome showed that patients with CKD were more likely to have WMH in the brain compared to the normal controls. Another meta-analysis showed a statistically significant decline in renal function in patients with moderate to severe WMH compared with those with no to mild WMH.

**Conclusions:**

The findings indicated that patients with CKD were more likely to experience WMH than demographically matched controls. On the other hand, patients with moderate to severe WMH in the brain had poor renal function more frequently than those with no to mild WMH.

## Introduction

Chronic kidney disease (CKD) is defined as abnormalities of the kidney structure or function present for >3 months (1). The prevalence and incidence of CKD have increased in recent years (2). CKD is associated with increased all-cause and cardiovascular mortality and a group of large vessel diseases, such as hypertension, arterial stiffness, and ischemic heart disease (3). There is an inverse relationship between CVD risk and glomerular filtration rate (GFR), independent of age, sex, and other risk factors (4, 5).

Many studies have found that patients with poor renal function are not only associated with large vessel disease but also with a higher prevalence of white matter hyperintensities (WMH), lacunes, and cerebral microbleeds (CMBs), especially the total burden of cerebral small vessel disease (cSVD) (6–8). At the same time, previous studies have shown that the overall burden of cSVD had a strong link with cognitive impairment and significantly affected the quality of life, exhibited poor health literacy, showed low adherence to medication, and showed greater morbidity and mortality rates in patients with CKD (3, 9, 10). However, as a primary marker of cSVD, previous studies on the associations between WMH and CKD are still conflicting (11–13). Currently, most reviews have described the relationship between CKD and cSVD, and few systematic reviews and meta-analyses have focused explicitly on the relationship between CKD and WMH. Therefore, our study aimed to conduct a systematic review of all the available studies on this topic and a meta-analysis of the association between WMH and CKD among observational studies.

## Materials and Methods

As this study was a systematic review of published studies, ethical approval was exempted by the Institutional Review Board of the Office of Research Administration, the Affiliated Jiangning Hospital of Nanjing Medical University, and informed patient consent was not required.

### Search Strategy

We collected the relevant clinical data from the literature published until 15 November 2020. All available studies in PubMed, Embase, Web of Science, the Cochrane Library, and Google Scholar were examined. This review focused on observational studies of the association between white matter hyperintensities and chronic kidney disease. We used the following medical subject headings and terms, without language restrictions, in conjunction with a highly sensitive search strategy: (chronic kidney disease) OR (CKD) OR (kidney function) OR (kidney failure) OR (renal disease) OR (renal insufficiency) OR (renal failure) OR [glomerular filtration rate (GFR)] OR [estimated glomerular filtration rate (eGFR)] OR (creatinine) OR (albuminuria) OR (microalbuminuria) OR (macroalbuminuria) OR (proteinuria) OR (kidney injury) AND (white matter hyperintensity) OR (white matter lesions) OR (white matter disease) OR (cerebral small vessel disease) OR (leukoencephalopathy) OR (leukoaraiosis) OR (demyelination of white matter).

The retrieval strategy was adjusted accordingly to different databases due to the dissimilarities of retrieval rules. Where there were multiple publications from the same study population, the most correlative article that reported sufficient data for the analysis was selected.

### Inclusion and Exclusion Criteria

Studies fulfilling all of the following criteria were included: (1) observational studies, including cross-sectional, longitudinal, cohort, and case-control studies; (2) studies involving patients 18 years or older, with evidence of kidney injury for at least three months; (3) studies of the association between CKD and WMH as the primary or secondary outcome; and (4) the results of studies presented as continuous or two-class data or those with insufficient data to calculate these statistics. The exclusion criteria were as follows: (1) studies on special kidney injury or white matter lesions, such as hereditary renal disease, demyelinating encephalopathy, metabolic encephalopathy, and toxic encephalopathy and (2) studies such as case reports, letters, and review articles, which were dismissed after the screening. The titles and abstracts of all the relevant items were screened to determine their eligibility for inclusion. The full articles were then checked for further eligibility as per the detailed inclusion criteria.

### Quality Assessment

The risk of bias was assessed by two independent reviewers using the Cross-Sectional/Prevalence Study Quality (CSSQ) scale recommended by the Agency for Healthcare Research and Quality (AHRQ). Two authors assessed the methodological quality of all the selected studies. The CSSQ contains the following eleven items in the form of questions: (1) define the source of information (survey, record review); (2) list the inclusion and exclusion criteria for exposed and unexposed participants (cases and controls) or refer to previous publications; (3) indicate the time period used for identifying patients; (4) indicate whether participants were consecutive, if not population-based; (5) indicate if the evaluators of the subjective components of the study were masked to other aspects of the status of the participants; (6) describe any assessment undertaken for quality assurance purposes (e.g., test/retest of primary outcome measurements); (7) explain any patient exclusion from the analysis; (8) describe how confounding data was assessed and/or controlled; (9) if applicable, explain how missing data was handled in the analysis; (10) summarize patient response rates and the completeness of the data collection; and (11) clarify what follow-up, if any, was expected and the percentage of patients for which incomplete data or follow-up was obtained. Each item was responded to with “yes,” “no,” or “unclear.” When any item of the CSSQ was not reported, a zero score was allocated. The total scores ranged from 0 to 11 (with 11 being the highest level of quality). Additionally, two reviewers assessed the studies independently based on the inclusion criteria.

### Data Extraction

We used a standardized data form in duplicate to collect the following descriptive information: surname and initials of the first author, the year of publication or submission, number of participants, study design, and population characteristics (age, sex (male/female), evaluation of kidney dysfunction, and white matter hyperintensities). We extracted or calculated the number of white matter hyperintensity signals in patients with or without renal insufficiency. For eGFR, the mean and standard deviation were extracted to calculate effect sizes.

### Statistical Analysis

For all studies, only the baseline data were used. The meta-analysis was divided into two parts. First, we calculated the combined OR value for the combined analysis of white matter hyperintensity in patients with or without renal insufficiency. For eGFR, the pooled mean and standard deviation were calculated for meta-analysis. The two meta-analyses were performed using the fixed-effects method or the random-effects method, depending on the absence or presence of significant heterogeneity. Because this review included only observational studies, a greater level of heterogeneity was expected. We used a funnel plot to check for publication bias, and sensitivity and subgroup analyses were undertaken to analyze the source of heterogeneity. The statistical heterogeneity among trials was assessed using the *I*^2^ index, with a *p* < 0.10 or *I*^2^ value > 50% being considered heterogeneous. A subgroup analysis was conducted to analyze heterogeneity. All statistical comparisons were two-tailed, and a *p* < 0.05 was considered statistically significant. All analyses were performed using STATA software (version 12.0).

### Literature Search

**Figure 1:**
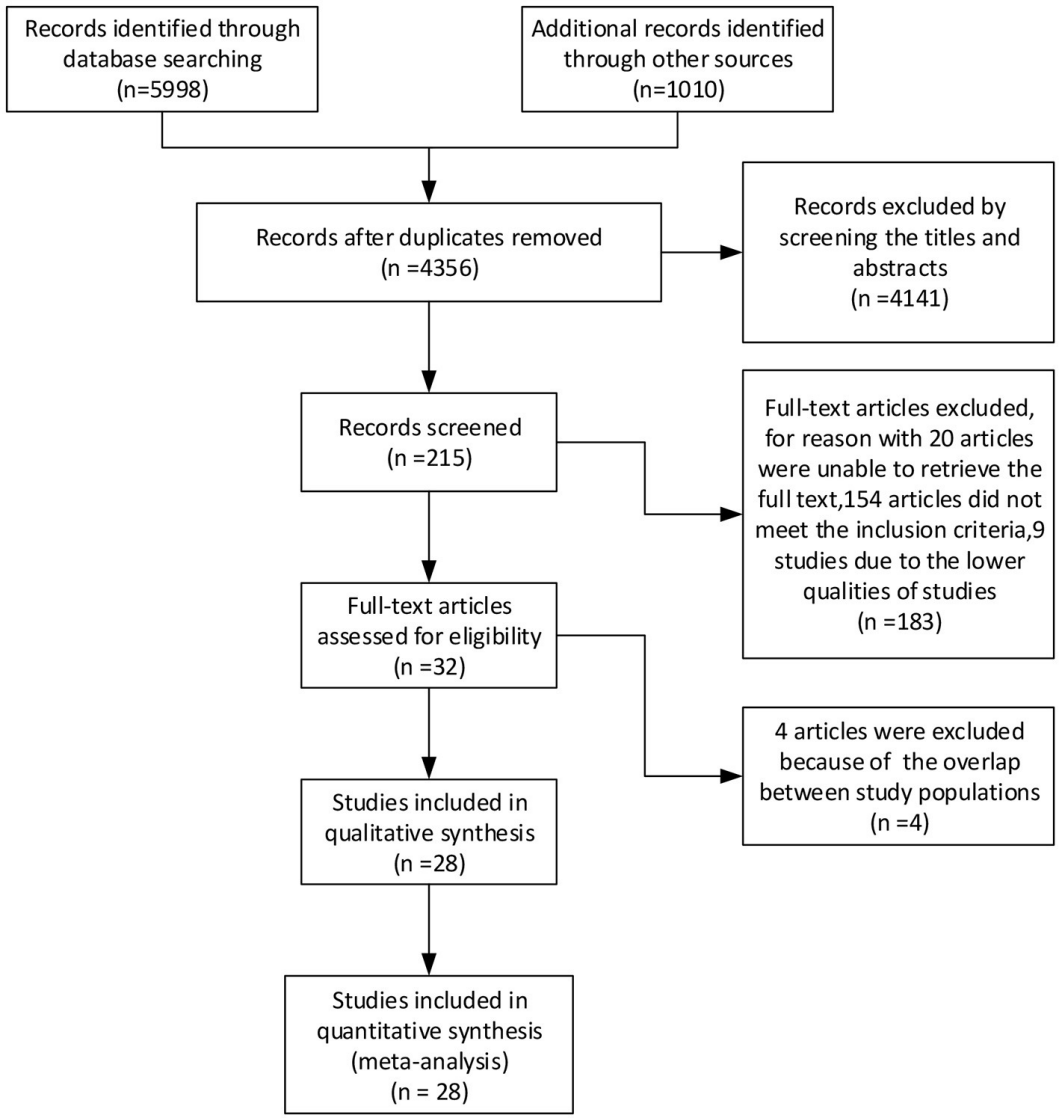
A PRISMA flow diagram for the derivation of studies included in the analyses.

The literature search yielded 7,008 articles, including 3,565 articles retrieved by PubMed, 1,524 articles from Embase, 35 clinical trials from the Cochrane Library, 874 articles from Web of Science, and 1,010 articles from Google Scholar, up until 18 October 2020. After screening the titles and abstracts and removing duplicate articles, 215 articles were included. The full text of these 215 articles were then searched for further analysis, and 183 were excluded because we could not retrieve the full text of 20 articles, 154 articles did not meet the inclusion criteria, and nine studies were of poor quality. Finally, four more articles were excluded because of the potential overlap between study populations. After analysis, 28 studies were included in the review. A flow diagram of the search strategy is provided in Figure 1.

## Results

### Description of Included Studies

The characteristics of the 28 studies are presented in Table 1. There were a total of 19,420 participants across 21 cross-sectional studies, four cohort studies, two case-control studies, and one longitudinal study. The mean age of the participants ranged from 44.5 to 79.0 years, and the participants were primarily normal individuals who came for health checkups; patients with stroke, those with type 2 diabetes, and those with CKD; local community participants; and voluntary participants; among others. In most studies, the number of male participants was more than female participants, except in eight studies. In three studies, the proportion of female participants was significantly higher than male participants (24, 35, 36). In the remaining five studies, the proportion of female participants was slightly higher than that of male participants. Glomerular filtration rate (GFR) <60 ml/(min·1.73m^2^) was used as the evaluation standard of CKD in most studies. The other evaluation criteria of CKD included urine protein, abnormally elevated urinary albumin-creatinine ratio (UACR), and serum creatinine levels. Unfortunately, in the study of Naganuma et al. (31), we could not obtain specific information for CKD evaluation. The Fazekas score is widely used to evaluate the severity of WMH, including periventricular WMH (PVWMH) and deep WMH (DWMH), as the scale showed good reliability in the intra-rater reliability analysis. Moreover, white matter lesion (WML) volume and other scales, such as a 10-point scale and Schmidt scale, have been used for measurements of WMH in some studies. Model adjustments generally included age, sex, arterial hypertension, dyslipidemia, diabetes mellitus, smoking, and obesity.

**Table 1:** Characteristics of included studies.

**Study**	**Study design**	**Participants**	**Mean age/year (SD)**	**Male n (%)**	**Evaluation of CKD**	**Measurements of WMH**	**Model adjustments**
Yang et al. (14).	Cross-sectional study	993 acute lacunar infarction patients	63.2 (11.4)	679 (68.4)	CKD GFR <60 ml/(min·1.73 m^2^) Urine protein negative (<25 mg/dL) positive (≥ 25 mg/dL)	Mild group, PVWMH/DWMH Fazekas score 0, 1; moderate-severe group, PVWMH/DWMH Fazekas score 2, 3.	Age, male, hypertension, diabetes, and hyperlipidemia, history of stroke, current smoker, and alcohol use.
Fandler-Hofler et al. (15).	Longitudinal study	101 stroke patients	60.2 (10.7)	74 (73.3)	Either eGFR <60 mL/min/1.73 m^2^ and/or albuminuria/ACR >30 mg/g).	Fazekas score 2–3.	Age, sex, arterial hypertension, dyslipidemia, diabetes mellitus, smoker, obesity, atrial fibrillation, coronary heart disease, history of stroke, and NIHSS at admission.
Yeh et al. (16).	Cohort study	87 patients with CKD and 50 controls	CKD 65.1 (9.0) Comparisons 62.6 (7.5)	CKD 58 (66.7) Comparisons 18 (36.0)	GFR <60 ml/(min·1.73 m^2^)	A 10-point scale (0–9).	Age, sex, educational level, FCRS score, CIRS score, and hemoglobin level.
Lau et al. (17).	Cross-sectional study	959 patients in China with ischemic stroke	69 (12.0)	576 (60.1)	GFR <60 ml/(min·1.73m^2^)	Fazekas scale	Age, sex, hypertension, hyperlipidemia, and diabetes mellitus, ever-smoking, and atrial fibrillation.
Xu et al. (12).	Cross-sectional study	126 primary intracerebral hemorrhage patients	61.8 (13.2)	71 (56.3)	GFR <60 ml/(min·1.73 m^2^) Urine protein negative (<15 mg/dL), trace (15–30 mg/dL), 1+ (30 mg/dL), 2+ (100 mg/dL), 3+ (300 mg/dL), 4+ (1,000 mg/dL)	Either confluent deep WMH (Fazekas score 2 or 3) or irregular periventricular WMH extending into the DWM.	Age, sex, hypertension, diabetes mellitus, previous ICH, previous stroke/TIA, smoker, alcohol, antiplatelets, antihypertensive drugs, and CRP.
Tsai et al. (18).	Cross-sectional study	142 patients with acute hypertensive intracerebral hemorrhage	GFR≥90 58.0 (24.0) 60≤GFR <90 60.0 (17.0) GFR <60 66.0 (20.0)	GFR≥90 25 (50.0) 60≤GFR <90 46 (64.8) GFR <60 18 (85.7)	Normal kidney function (GFR≥90), mild kidney disease (60≤GFR <90), moderate to severe kidney disease (GFR <60).	WMH is a signal abnormality of variable size in the white matter that shows hyperintensity on FLAIR and without cavitation. WMH is further categorized into periventricular and deep (basal ganglion and brain stem regions) according to the locations.	Age, male, diabetes mellitus, and hypertension.
Del Brutto et al. (11).	Cross-sectional study	314 community-dwelling older adults	71.1 (8.1)	130(41.1)	Normal kidney function (GFR≥90), mild kidney disease (GFR 60-90), moderate to severe kidney disease (GFR15-59).	WMH was defined as lesions appearing hyperintense on T2-weighted images that remained bright on FLAIR (without cavitation) and graded according to the modified Fazekas scale in none, mild, moderate and severe.	Age, sex, glucose levels, total cholesterol, education, blood pressure, and edentulism.
Suda et al. (19).	Cross-sectional study	284 consecutive acute ischemic stroke patients	72.2 (12.3)	177 (62.3)	Proteinuria group (UACR≥300 mg/g creatinine), microalbuminuria group (30.0 mg/g creatinine≤UACR≤300 mg/g creatinine), normal albuminuria group UACR <30.0 mg/g creatinine.	The degree of WML to Fazekas 0 to 1 (none-to-mild WML) and 2 to 3 (severe WML).	Age, male, BNP, and hypertension.
Hayashi et al. (20).	Cross-sectional study	1,716 participants aged 40–80 years, who received health checkups	61.8 (9.9)	1,175 (68)	GFR <60 ml/(min·1.73 m^2^)	WMH were classified into 5 grades (0–4) according to the Shinohara grading: grade 1 was considered mild, grades 2–3 as moderate, and grade 4 as severe WMH.	Age, sex, BMI, current smoking, hypertension, diabetes, dyslipidemia and eGFR category.
Jeon et al. (21).	Cohort study	1,138 acute ischemic stroke patients	73.3 (9.9)	156 (61)	GFR <60 ml/(min·1.73 m^2^)	Periventricular white matter hyperintensity (WMH) extending into the deep white matter (Fazekas grade 3) and confluence or early confluence of deep WMH (Fazekas grades 2–3) were considered to represent the presence of WML.	Age, sex, hypertension, diabetes, smoking, dyslipidemia, previous stroke, and previous CHD.
Cho et al. (13).	Cross-sectional study	1,215 cognitively normal individuals	Normal UACR 63.5 (7.6) Elevated UACR 65.8 (7.4)	Normal UACR 497 (43.6) Elevated UACR 89 (70.7)	Subjects were considered to have an abnormally elevated UACR (above microalbuminuria level) if the value was≥17 mg/g for men and≥25 mg/g for women	WMH visual rating scale was modified using the Fazekas scale.	Age, sex, history of hypertension, diabetes mellitus, hyperlipidemia, ischemic heart disease, stroke, fasting glucose, systolic blood pressure, diastolic blood pressure, total cholesterol level, body mass index, status of current smoking and alcohol drinking, educational level, and intracranial volume.
Zong et al. (22).	Cross-sectional study	1,632 patients with AIS or TIA	62.3 (11.5)	1,118 (68.5)	CKD stage1: eGFR≥90 ml/min per 1.73 m^2^, stage 2:60≤eGFR <90 ml/min per 1.73 m^2^, stage3:30≤eGFR <60 ml/min per 1.73 m^2^, stage4: 15≤eGFR <30 ml/min per 1.73 m^2^, stage5: eGFR <15 ml/min per 1.73 m^2^.	The Fazekas scale was used to score both the severity of periventricular WMH (PVH) and deep subcortical WMH (DWMH).	Age, male, smoking,diabetes mellitus, hypertension, hypercholesterolemia, atrial fibrillation,ischemic heart disease,and ischemic stroke/TIA.
Sink et al. (23).	Cross-sectional study	263 unrelated AAs with type 2 diabetes	60.4 (9.6)	98 (37.3)	eGFR <60 mL/min/1.73 m^2^ and/or UACR >30 mg/g	WML volume was determined by summing the binary lesion maps and multiplying by the voxel volume, and values are reported in cubic centimeters.	Age, education, sex, BMI, HbA1c level, and hypertension.
Toyoda et al. (24).	Cross-sectional study	1,937 neurologically normal individuals	59.4 (7.9)	100 (59.8)	CKD was defined as either positive proteinuria or eGFR <60 ml/min/1.73 m^2^	No details	Age and sex, hypertension and dyslipidemia, smoking and alcohol habit.
Saji et al. (25).	Cohort study	152 acute lacunar stroke patients	With CKD 77 (70–82) Without CKD 64 (59–71)	With CKD 29 (66) Without CKD 72 (67)	CKD was defined as an estimated glomerular filtration rate of <60 ml/min/1.73 m^2^ on admission	WMH was defined as an irregular periventricular hyperintensity (Fazekas grade≥3) and/or early confluent or confluent separate deep white matter hyperintense lesions (Fazekas grade≥2) on T2WI and FLAIR images.	Age, sex, body mass index and blood pressure.
Kuriyama et al. (26).	Case-control study	273 voluntarily participants who had participated in a hospital-based health check-up and underwent repeated brain MRI examinations	66.9 (6.1)	175 (64.1)	CKD was defined as estimated glomerular filtration rate (eGFR) under 60 ml/min/1.73 m^2^ in accordance with established criteria.	DWLs were graded semi-quantitatively from Grade 0 (none) to Grade 3 (severe) according to the Fazekas classification.	Age, sex, hypertension, diabetes mellitus, hyperlipidemia, ischemic heart disease, drinking and smoking.
Umemura et al. (27).	Cross-sectional study	79 type 2 diabetic patients	eGFR≥60 74.6 (5.3) eGFR <60 79.0 (6.7)	eGFR≥60 20 (37.0) eGFR <60 10 (40.0)	CKD was defined as the presence of albuminuria and/or a low estimated glomerular filtration rate (eGFR <60 ml/min/1.73 m^2^)	Periventricular WMLs were classified as grades 1–3 as follows: grade 1 (mild), pencil-thin lining; grade 2 (moderate), smooth halo, and grade 3 (severe), large confluence. Subcortical WMLs were classified according to the following three grades in accordance with the Fazekas scale: grade 1 (mild), punctuate foci; grade 2 (moderate), early confluence, and grade 3 (severe), diffuse confluence. A grade of ≥2 was regarded as advanced periventricular or subcortical WMLs.	Age, sex, education years, and diabetes duration.
Steinicke et al. (28).	Cross-sectional study	2,500 young (18–55 years) patients with first-ever ischemic stroke	44.5 (8.4)	1,512 (60.5)	eGFR was categorized in 3 groups: (1) under filtration: <60 mL/min; (2) normal filtration: 60 to 120 mL/min; (3) hyperfiltration: >120 mL/min.	WMHs were defined as lesions with high signal intensity on T2-weighted images in the absence of evidence for complete tissue destruction and were rated according to the Fazekas scale.	Age, men, NIHSS, diabetes, hypertension, cardiovascular disease, hyperlipidemia, regular alcohol consumption, higher alcohol consumption, current smoking, BMI.
Takahashi et al. (29).	Cross-sectional study	2,106 participants who underwent a brain checkup at HIMEDIC imaging center	eGFR ≥90 51 (10) eGFR 60–89 55 (9) eGFR≤60 63 (8)	eGFR ≥90 199 (54) eGFR 60-89 986 (65) eGFR≤60 183 (74)	Kidney function was classified into eGFR≥90, 60-89, or≤60mL/min/1.73 m^2^, based on the modified Kidney Disease Outcomes Quality Initiative clinical practice guidelines on chronic kidney disease.	DSWMH was classified as follows: grade I, punctate or discrete foci≤3 mm in diameter; grade II, punctate or discrete foci>3 mm in diameter; grade III, confluent foci; grade IV, confluence widely distributed throughout the white matter. PVH was also classified into 4 grades: grade I, frontal or occipital caps of the lateral ventricles; grade II, smooth halo along the whole periventricular area; grade III, irregular PVH extending partially into the deep white matter; and grade IV, extending throughout the deep and subcortical white matter.	Age and sex.
Naganuma et al. (30).	Case-control study	179 HD patients and 58 healthy control subjects	HD patients 58.2 (13.6) Healthy subjects 55.9 (9.5)	HD patients 114 (63.7) Healthy subjects 31 (53.4)	No details	These WMHs were classified into PVH and DSWMH according to the classification of Fazekas. PVH was categorized as: grade 0, absent; grade 1, caps or pencil-thin lining; grade 2, smooth halo; and grade 3, irregular PVH extending into deep white matter. DSWMH was categorized as: grade 0, absent; grade 1, punctuate foci; grade 2, beginning confluence of foci; and grade 3, large confluent areas.	Gender, hypertension, diabetes mellitus, dyslipidemia, and current smoking.
Shima et al. (31).	Cross-sectional study	324 CKD patients	Patients without PVH 51.6 (17.0) Patients with PVH 68.5 (10.2)	Patients without PVH 82 (54.7) Patients with PVH 110 (63.2)	CKD was defined as patients with kidney damage, as reported by KDIGO	The severity of PVH was classified according to Fazekas as follows: grade 0, absent; grade 1, caps or pencil-thin lining; grade 2, smooth halo; grade 3, irregular and extending into the deep white matter.	Age, gender, diabetes, anticoagulation or antiplatelet, therapy, LDL cholesterol, hemoglobin, systolic pressure, diastolic pressure, and pulse pressure.
Ueda et al. (32).	Cross-sectional study	202 consecutive patients with ischemic stroke	71.98 (-)	126 (62.4)	Kidney function was classified into eGFR≥60, or <60 mL/min/1.73 m^2^	Two groups (Grade 1 or more and less than Grade 1) based on the classification of Fazekas.	Age, male, diabetes, hypertension, hyperlipidemia, smoking, atrial fibrillation, past history of ischemic stroke, and past history of ischemic heart disease.
Oksala et al. (33).	Cohort study	378 patients with acute stroke	70.7 (7.6)	181 (47.9)	Patients were divided into those with normal or mildly impaired (Stages 1 to 2; eGFR ≥60 mL/min/1.73 m^2^);eGFR and those with low to moderate (Stage 3; eGFR <60 mL/min/1.73 m^2^).	WMLs were rated on proton density-weighted images in accordance with the Leukoaraiosis and Disability (LADIS) rating in older adults as no to mild, moderate, and severe degree.	Age, sex, stroke severity, hypertension, diabetes mellitus, AF, HF, MI, PAD, smoking, and serum cholesterol.
Otani et al. (34).	Cross-sectional study	1,008 participants from a general population of Ohasama	66.4 (5.7)	330 (32.74)	CCr <60 ml/min/1.73 m^2^	WMHs were defined as hyperintensities only on T2-weighted images, and they were graded according to Fazekas as follows: absent (grade 0), punctate (grade 1), early confluent (grade 2) and confluent (grade 3).	24-h systolic BP, sex, age, BMI ≥25, ever smoker, ever drinker, antihypertensive, medication, hypercholesterolemia, diabetes, history of heart disease.
Weiner et al. (35).	Cross-sectional study	335 participants	73.4 (8.1)	89 (26.6)	The presence of micro- or macroalbuminuria or non-albuminuric participants.	WMH severity was graded by a 10-point scale, successively increasing from no or barely detectable change (grades 0 and 1, respectively) to almost all white matter involved (grade 9).	Age, center and education, diabetes, cardiovascular disease, use of angiotensin converting enzyme (ACE) inhibitors or angiotensin receptor blockers, sex, race, hypertension.
Wada et al. (36).	Cross-sectional study	625 individuals in the local community	Grade 0 65.2 (5.0) Grade 1 69.1 (4.2) Grade 2 70.1 (3.8) Grade 3 70.5 (2.8)	Grade 0 73 (41.2) Grade 1 137 (46.4) Grade 2 42 (42.9) Grade 3 28 (50.9)	CKD was defined as a urinary albumin-creatinine ratio of >30 mg/g or an estimated glomerular filtration rate (eGFR) of <60 ml/min per 1.73 m^2^.	WML was defined as at least one focal lesion in cerebral white matter with corresponding hyperintensity on FLAIR images. the Fazekas scale was used to score WMLs.	Age, sex, TC/HDL cholesterol ratio, diabetes, current smoker, and maximal IMT.
Anan et al. (37).	Cross-sectional study	192 subjects for the treatment of type 2 DM detected on medical examination	WML-negative 57 (6) WML-positive 57 (8)	WML-negative 56 (54) WML-positive 34 (50)	Urinary albumin excretion levels were used to define three categories of albuminuria: <30 mg/24 h, normoalbuminuria; 30–299 mg/24 h, microalbuminuria; and ≥300 mg/24h, macroalbuminuria.	(1) normal scans (WMLs absent), if there was either absent or only slight periventricular hyperintensity (small caps or pencil-thin lining), fewer than five focal lesions, and no confluent lesions; or (2) WMLs present, if there was moderate or severe periventricular hyperintensity, five or more focal lesions, or confluent lesions.	None
Martinez-Veaet al. (38).	Cross-sectional study	52 patients without diabetes with CKD (stage 3 or 4) aged 30 to 60 years	White Matter Lesion Positive 54.4 (5.3) White Matter Lesion Negative 46.3 (9.1)	White Matter Lesion Positive 10 (58.8) White Matter Lesion Negative 28 (80.0)	All patients were aged between 30 and 60 years and had CKD, with serum creatinine levels > 2.03 mg/dL and <7.91 mg/dL.	We classified our patients as having white matter lesions with the Fazekas and Schmidt scale if there was: (1) irregular periventricular hyperintensity extending into deep white matter or marked areas of hyperintensity completely surrounding the lateral ventricles, or (2) early confluent or large confluent areas or more than 5 focal lesions at some distance from the ventricles.	Age, systolic blood pressure, LVM index, vascular nephropathy, and CRP.

*WMH, white matter hyperintensities; PVH, periventricular WMH; DWMH, deep subcortical WMH; DWM, deep white matter; GFR, glomerular filtration rate; eGFR, estimated glomerular filtration rate; ACR, Albumin-Creatinine Ratio; UACR, Urinary Albumin-Creatinine Ratio; WMLs, white matter lesions; DWLs, deep white matter lesions; HD, hemodialysis patients; CCr, Calculated creatinine clearance; AIS, acute ischemic stroke; TIA, transient ischemic attack; AAs, African Americans; DM, diabetes medications; ICH, intracerebral hemorrhage; CRP, C-reactive protein; BNP, brain natriuretic peptide; BMI, body mass index; CHD, coronary heart disease; NIHSS, National Institutes of Health Stroke Scale; AF, atrial fibrillation; HF, heart failure; MI, myocardial infarction; PAD, peripheral artery disease; TC, total cholesterol; HDL, high-density lipoprotein; LDL, low-density lipoprotein; IMT, intima-media thickness; ACE, angiotensin-converting enzyme; LVM, left ventricular mass*.

Twenty-eight studies met the inclusion criteria, including twenty-one cross-sectional studies, four cohort studies, two case-control studies, and one longitudinal study. Eleven studies had more than 500 participants. The number of participants in eight studies exceeded 1,000, and only two studies had <100 participants. In general, most of the included studies showed that chronic kidney disease was associated with increased severity of WMH; furthermore, the severity of kidney dysfunction was positively correlated with the severity of WMH (11, 16, 17, 21, 23–26, 28–30, 33–38). However, there was no significant correlation between GFR level and WMH severity in three studies (19, 20, 27). In the cross-sectional study by Lei Yang and colleagues (14), 993 patients with acute lacunar infarction aged 25–95 years were enrolled to assess kidney function and the severity of PVWMH and DWMH. They found that CKD was independently associated with moderate-severe PVWMH in patients with acute lacunar infarction but not with DWMH. Similarly, the study performed by Zong et al. demonstrated that renal dysfunction (eGFR) was independently associated with the severity of PVH but not of subcortical DWMH (SDWMH) in patients with acute ischemic stroke (22). However, another cross-sectional study with 142 patients with acute hypertensive intracerebral hemorrhage (ICH) arrived at controversial conclusions: In patients with hypertensive ICH, the prevalence of CKD was associated with the prevalence of cerebral microbleeds (CMB) and DWMH but not PVWMH. A large cross-sectional study with 959 patients with ischemic stroke in China showed that renal impairment was independently associated with microbleed, white matter hyperintensity, and global SVD burden in individuals aged below 60 but not in those aged 60 and above. However, another community-based study of older adult individuals in Japan, with an average age ranging from 65.2 to 70.5 years, demonstrated that patients with worse kidney function tended to have more lacunar infarcts and higher grades of white matter lesions. In addition, the mean grades of WMLs or the mean numbers of lacunar infarctions were greater in patients with albuminuria than in those without albuminuria.

### Meta-Analyses

**Figure 2:**
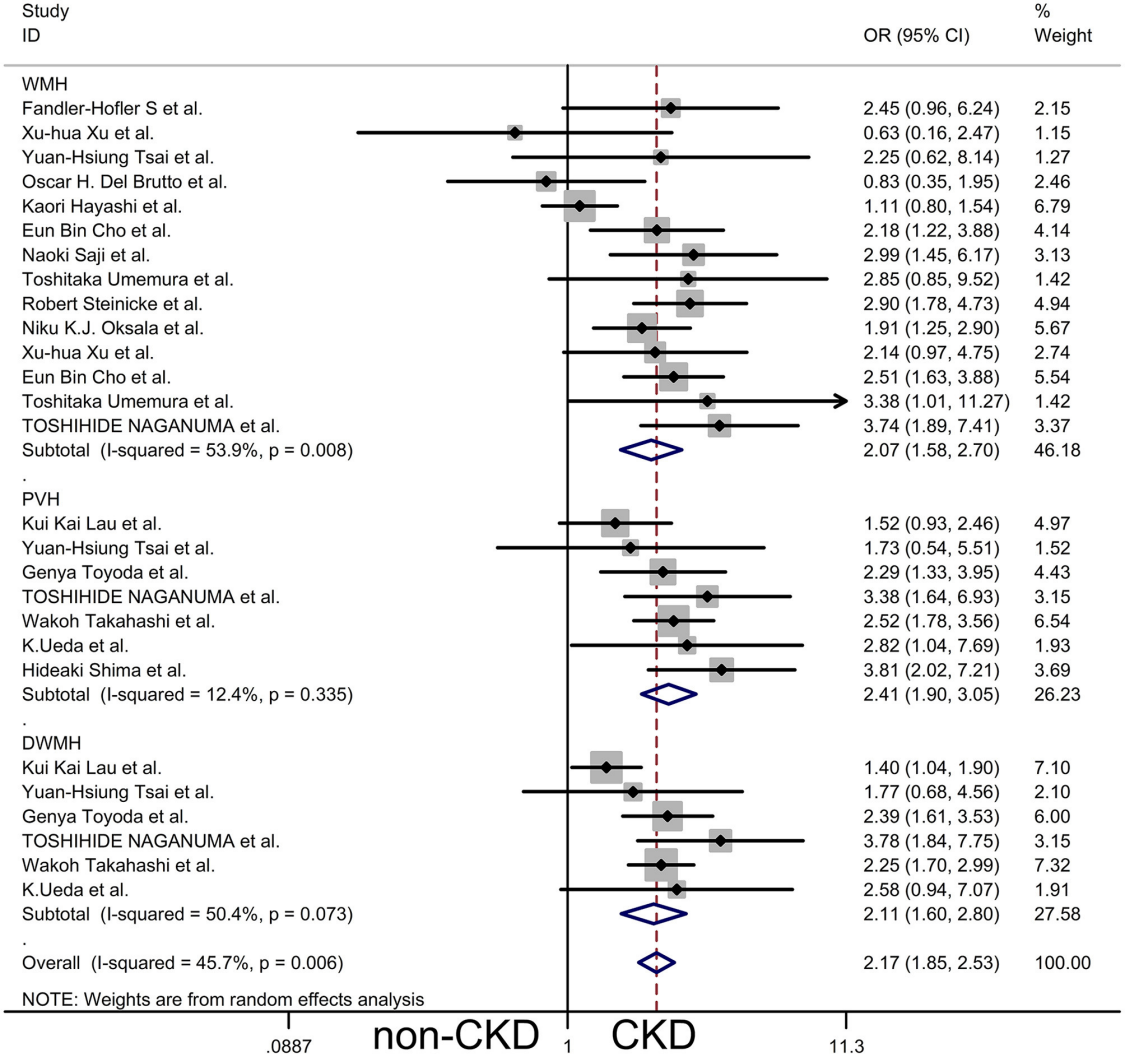
The subgroup analysis of a forest plot of comparison of CKD vs. non-CKD.

Most of the studies we included were divided into two groups according to whether there was CKD or not; therefore, we performed a meta-analysis of the presence of WMH in CKD vs. non-CKD (12 cross-sectional studies, two cohort studies, one case-control study, and one longitudinal study). The remaining studies were used as a reference for the meta-analysis outcome, and baseline data were extracted from all studies for meta-analysis. We pooled ORs for the presence of WMH, PVH, and DWMH of CKD vs. non-CKD by subgroup analysis, and a random-effects model was used to compare the differences (Figure 2). The results showed that compared to patients without CKD, the overall risk of WMH in CKD patients was OR 2.17, 95% CI [1.85, 2.53], *z* = 9.74, *p* = 0.000. However, the results showed bias because a few studies were included in the meta-analysis more than once. Consequently, the results of the subgroup analysis were closer to those of the general population, WMH OR 2.07, 95% CI [1.58, 2.70], *z* = 5.35, *p* = 0.000, PVH OR 2.41, 95% CI [1.90, 3.05], *z* = 7.30, *p* = 0.000, DWMH OR 2.11, 95% CI [1.60, 2.80], *z* = 5.22, *p* = 0.000. The main outcome showed that patients with CKD were more likely to have WMH in the brain compared to the normal controls, as shown by the outcomes in Figure 2. Although there was a statistically significant difference in WMH between patients with CKD and those without CKD, this difference showed significant heterogeneity, and the results of *I*^2^ and P-values in the three subgroups were WMH *I*^2^ = 59.3%, *p* = 0.008; PVH *I*^2^ = 12.4%, *p* = 0.335; and DWMH *I*^2^ = 50.4%, *p* = 0.073. This was probably due to the different methods of assessing CKD and WMH, the different demographic characteristics, and the different complications in each study.

**Figure 3:**
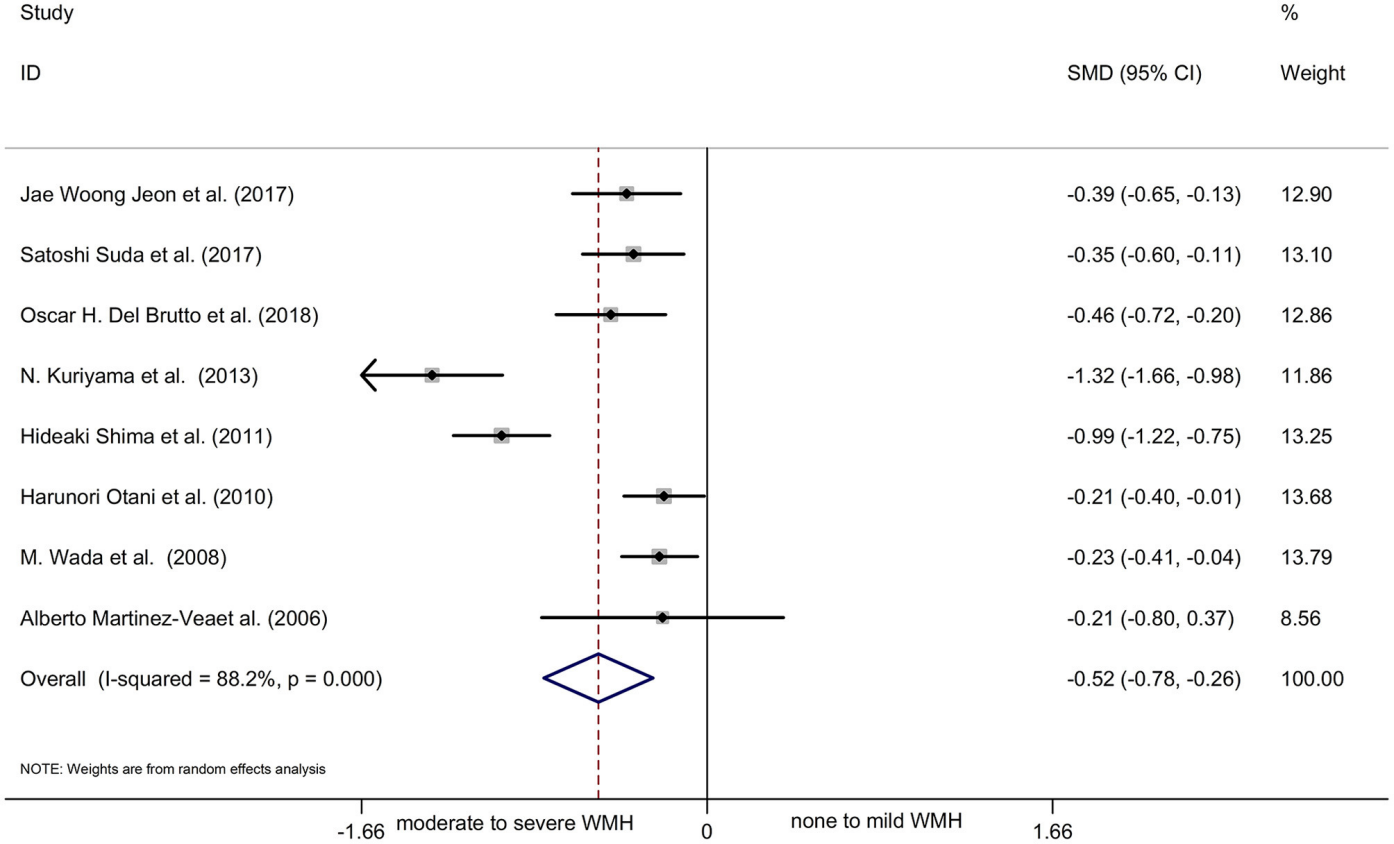
A forest plot of comparison of none-to-mild WMH vs. moderate-to-severe WMH.

**Figure 4:**
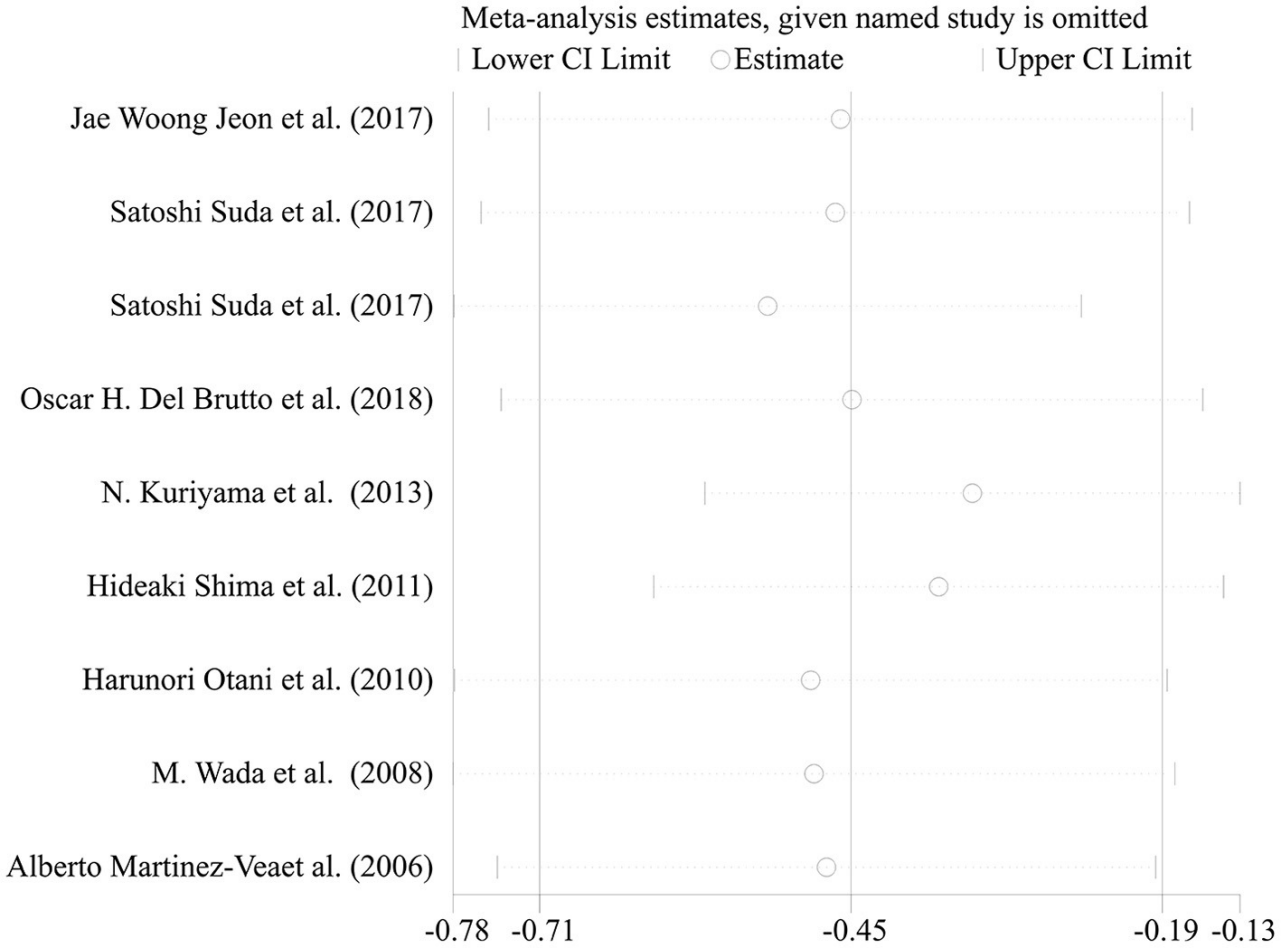
Sensitivity analysis of comparison of none-to-mild WMH vs. moderate-to-severe WMH.

Most of the remaining studies were grouped according to the severity of WMH with the evaluation of the Fazekas scale; grade 0–1 was considered none-to-mild, and grades 2–3 were considered moderate-to-severe WMH. Due to the diverse grouping methods, some results were combined with the mean and standard deviation before the meta-analysis. The following formulae were used to calculate the combined mean and standard deviation: the combined average X = (x1^*^n1 + *x*2^*^n2)/(n1+n2), where x and n are the number and average of their respective groups, and the combined variance *S*^2^ = ((n1-1)^*^S1^2^ + (*N*2−1)^*^S2^2^)/(n1+N2-1), where S is the standard deviation of each group and *S*^2^ is the variance of each group. The six cross-sectional studies, one cohort study, and one case-control study were pooled and analyzed using a random-effects model. This analysis showed a statistically significant decline in renal function in patients with moderate-to-severe WMH than in those with none-to-mild WMH (Figure 3: SMD = −0.52, 95% CI [-0.78,−0.26], *z* =3.89, *p* = 0.000, df = 7). The results showed significant heterogeneity because the *I*^2^ and P-values were 88.2 and 0.000, respectively (Figure 3). Therefore, sensitivity analysis with a random-effects model was used to explore the sources of heterogeneities (Figure 4). As shown in Figure 4, the sensitivity analysis results comparing none-to-mild WMH vs. moderate-to-severe WMH demonstrated no significant difference after the removal of each study one by one.

### Publication Bias

**Figure 5:**
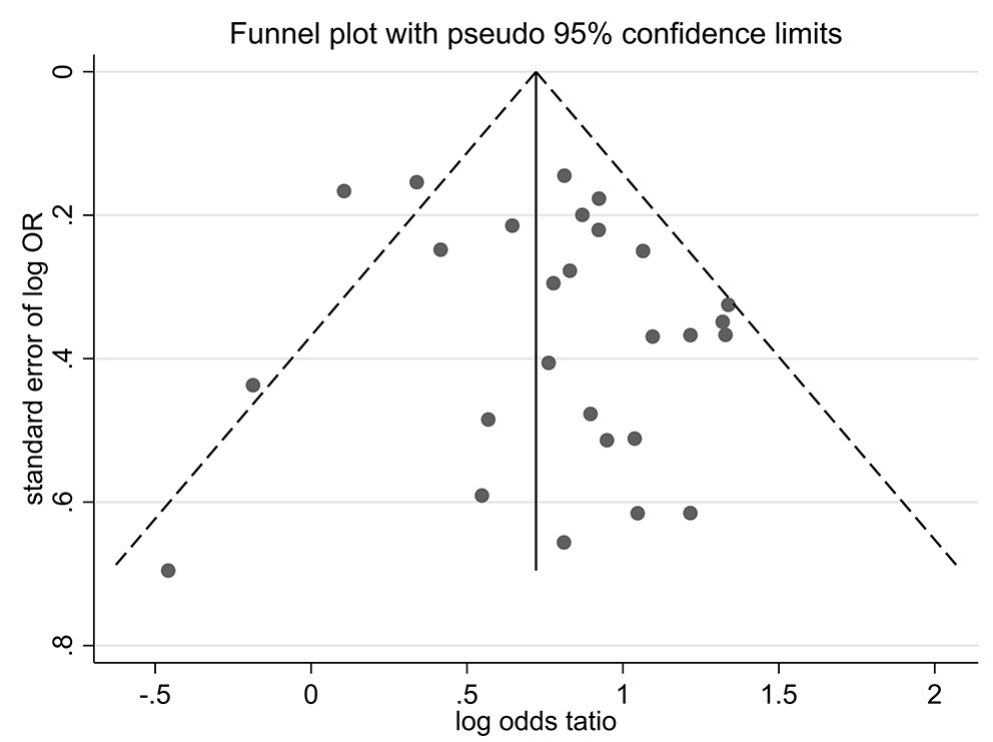
A funnel plot of comparison of CKD vs. non-CKD.

**Figure 6:**
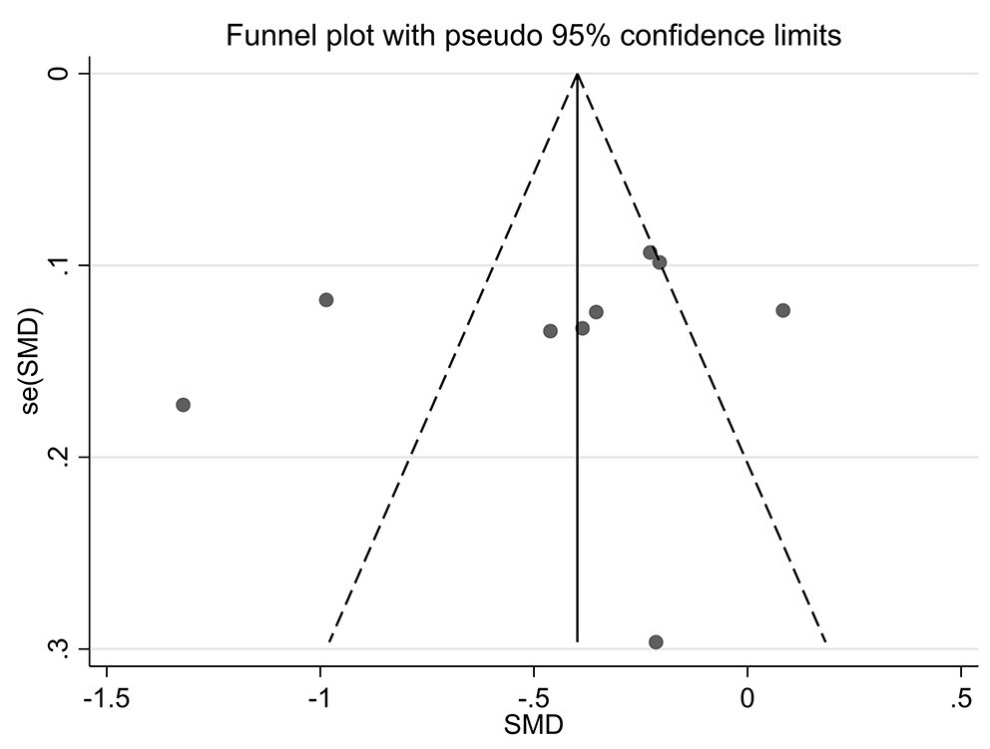
A funnel plot of comparison of none-to-mild WMH vs. moderate-to-severe WMH.

Funnel plots of effect size vs. standard error were created using STATA software (version 12.0) to investigate possible publication bias. No significant asymmetry was observed in the comparative funnel plots between patients with CKD and non-CKD patients (Begg's test for the effects: Kendall's score (P-Q) = 87, Std. Dev. of score = 47.97, *z* = 1.81, *p* = 0.07, Figure 5). Similarly, we did not find significant statistical asymmetry in the funnel plots comparing none-to-mild WMH with moderate-to-severe WMH (Begg's test for the effects: Kendall's score (P-Q) = −12, Std. Dev. of score = 9.59, *z* = −1.25, *p* = 0.21, Figure 6).

## Discussion

Chronic kidney disease is a global public health issue worldwide. The complications caused by CKD, especially abnormalities in brain structure, have increased the attention it receives as the risk factors for CKD, such as hypertension, diabetes, and obesity have increased along with a longer life span of patients with CKD (2). The progress of magnetic resonance imaging (MRI) techniques has brought many new perspectives for assessing brain structure and function in patients with CKD (39, 40). WMHs are more frequent (up to 70% in patients requiring dialysis) in patients with CKD than in those without CKD or even in the early stage of CKD, suggesting that structural alterations begin early in the CKD disease process (36, 41). The white matter plays an important role in coordinating interactions between different regions of and in the normal functioningof the brain, and impaired white matter integrity appears to be a primary contributor to cognitive decline in patients with CKD (42–45).

The review included 19,420 participants and revealed that most previous observational studies were related to this topic. The results showed that patients with CKD had a higher risk of WMH than those without. In addition, the results of subgroup analysis showed that the severity of PVH and DWMH presented significant differences in patients with or without CKD. Another meta-analysis that compared renal function in patients with moderate-to-severe WMH with those with none-to-mild WMH showed that the patients with moderate-to-severe WMH presented a more serious decrease in kidney function.

The mechanism for the impairment of WMH in patients with CKD is thought to be associated with cerebrovascular disease, systemic inflammation, cerebral hypoperfusion, oxidative stress, disturbances of small blood vessels, breakdown of the blood-brain barrier (BBB), glial activation, loss of oligodendrocytes, and demyelination caused by chronic diffuse hypoperfusion (46–48). Cerebrovascular disease is a significant risk factor for the development of WMH in patients with CKD. The vascular risk factors relevant to the impairment of WMH in the brain likely include hypertension, diabetes, hyperlipidemia, elevated homocysteine, and cigarette smoking (49, 50). Hypertension was a primary vascular risk factor, as reported in previous studies. Mean arterial pressure was associated with white matter hyperintensity volume even in the absence of associations between changes in the brain tissue and tonometry measures (51, 52). The kidney and the brain are often involved simultaneously when vascular risk factors are present. The principal reason is that the kidney and the brain share unique susceptibilities to vascular injury since the vascular regulation of the microvasculatures of the two organs is anatomically and functionally similar. Moreover, the arteries of the kidney and brain are exposed to high pressure, and they have to maintain a strong vascular tone to provide a large pressure gradient over a short distance; therefore, hypertensive vascular damage occurs first and most severely in such strained vessels (52, 53).

In conclusion, the systematic review and meta-analysis findings indicate that patients with CKD are more likely to experience WMHs (including PVHs and DWMHs) than demographically matched controls. On the other hand, patients with moderate-to-severe WMH in the brain have poor renal function more frequently than those with no to mild WMH.

### Study Strengths and Limitations

▸ A strength of this systematic review is that it analyzes the association between CKD and WMH concerning almost all the relevant observational studies available in the public domain and meta-analyses is that it was carried out from the perspective of CKD and WMH.
▸ The main limitation of the present study is that the meta-analysis is based mostly on cross-sectional studies because most relevant studies are cross-sectional. Additionally, we did not compare the severity of PVH and DWMH because of the different pathogenesis.
▸ Another limitation is that we used the raw data to compare differences, which may be one of the sources of heterogeneity in this study.

## Data Availability Statement

The original contributions presented in the study are included in the article/Supplementary Material, further inquiries can be directed to the corresponding authors.

## Author Contributions

X-MC and J-RL contributed to the conception, design, and manuscript revision. C-YY, LW, and J-YJ contributed to the selection process and screening of trials included in this metaanalysis. QD and C-SW contributed to data extraction and risk of bias assessment. C-SW and C-YY contributed to the data analysis. RZ and X-RY were involved in the writing of the paper. All authors contributed to the article and approved the submitted version.

## Funding

This study was funded by the clinical medical science and technology development fund of Jiangsu University (JLY2021153), Nanjing Medical Science and technique Development Foundation (YKK20203), and the Project of Development Research of Kangda College in Nanjing Medical University (KD2019KYJJYB047).

## Conflict of Interest

The authors declare that the research was conducted in the absence of any commercial or financial relationships that could be construed as a potential conflict of interest.

## Publisher's Note

All claims expressed in this article are solely those of the authors and do not necessarily represent those of their affiliated organizations, or those of the publisher, the editors and the reviewers. Any product that may be evaluated in this article, or claim that may be made by its manufacturer, is not guaranteed or endorsed by the publisher.

## Abbreviations

CKD: chronic kidney disease
WMH: white matter hyperintensities
CMBs: cerebral microbleeds
cSVD: cerebral small vessel disease
CSSQ: Cross-Sectional/Prevalence Study Quality scale
AHRQ: Agency Healthcare Research and Quality
PVH: periventricular WMH
DWMH: deep subcortical WMH
DWM: deep white matter
GFR: glomerular filtration rate
eGFR: estimated glomerular filtration rate
ACR: albumin-creatinine ratio
UACR: urinary albumin-creatinine ratio
WMLs: white matter lesions
DWLs: deep white matter lesions
HD: hemodialysis patients
KDIGO: Kidney Disease: Improving Global Outcomes
CCr: Calculated creatinine clearance
AIS: acute ischemic stroke
TIA: transient ischemic attack
AAs: African Americans
DM: diabetes medications
ICH: intracerebral hemorrhage
CRP: C-reactive protein
BNP: brain natriuretic peptide
BMI: Body Mass Index
CHD: Coronary heart disease
NIHSS: National Institutes of Health Stroke Scale
AF: atrial fibrillation
HF: heart failure
MI: myocardial infarction
PAD: peripheral artery disease
TC: total cholesterol
HDL: high-density lipoprotein
LDL: low-density lipoprotein
IMT: intima-media thickness
ACE: angiotensin-converting enzyme
LVM: Left ventricular mass
SMD: standardized mean difference
CI: confidence interval
OR: odds ratio
BBB: blood–brain barrier.
