# Supplementary material for: Association Between White Matter Hyperintensities and Chronic Kidney Disease: A Systematic Review and Meta-Analysis
Source: Front Med (Lausanne). 2022 May 3;9:770184. doi: 10.3389/fmed.2022.770184 (PMC9112853; doi:10.3389/fmed.2022.770184)
Supplement: Supplementary file 1 [file Data_Sheet_1.docx]

CKD, chronic kidney disease OR CKD OR kidney function OR kidney failure OR renal disease OR renal insufficiency OR renal failure OR glomerular filtration rate (GFR) OR estimated glomerular filtration rate(eGFR) OR creatinine OR albuminuria OR microalbuminuria OR macroalbuminuria OR proteinuria OR kidney injury

WMH, White matter hyperintensity OR White matter lesions OR white matter disease OR cerebral small vessel disease OR Leukoencephalopathy OR Leuko-araiosis OR Demyelination of white matter

Pubmed, (((((((((((((((chronic kidney disease) OR (CKD)) OR (kidney function)) OR (kidney failure)) OR (renal disease)) OR (renal insufficiency)) OR (renal failure)) OR (glomerular filtration rate (GFR))) OR (estimated glomerular filtration rate(eGFR))) OR (creatinine)) OR (albuminuria)) OR (microalbuminuria)) OR (macroalbuminuria)) OR (proteinuria)) OR (kidney injury)) AND (((((((White matter hyperintensity) OR (White matter lesions)) OR (white matter disease)) OR (cerebral small vessel disease)) OR (Leukoencephalopathy)) OR (Leuko-araiosis)) OR (Demyelination of white matter))

Embase, ("chronic kidney disease" or CKD or "kidney function" or "kidney failure" or "renal disease" or "renal insufficiency" or "renal failure" or "glomerular filtration rate" or "estimated glomerular filtration rate" or creatinine or albuminuria or microalbuminuria or macroalbuminuria or proteinuria or "kidney injury") and ("White matter hyperintensity" or "White matter lesions" or "white matter disease" or "cerebral small vessel disease" or Leukoencephalopathy or "Leuko-araiosis" or "Demyelination of white matter")

Cochrane Library,("chronic kidney disease" or CKD or "kidney function" or "kidney failure" or "renal disease" or "renal insufficiency" or "renal failure" or "glomerular filtration rate" or "estimated glomerular filtration rate" or creatinine or albuminuria or microalbuminuria or macroalbuminuria or proteinuria or "kidney injury") and ("White matter hyperintensity" or "White matter lesions" or "white matter disease" or "cerebral small vessel disease" or Leukoencephalopathy or "Leuko-araiosis" or "Demyelination of white matter")

Web of Science

TS=("White  matter  hyperintensity"  or  "White  matter  lesions"  or  "white  matter  disease"  or  "cerebral  small  vessel  disease"  or  Leukoencephalopathy  or  "Leuko-araiosis"  or  "Demyelination  of  white  matter")  and TS=("chronic  kidney  disease"  or  CKD  or  "kidney  function"  or  "kidney  failure"  or  "renal  disease"  or  "renal  insufficiency"  or  "renal  failure"  or  "glomerular  filtration  rate"  or  "estimated  glomerular  filtration  rate"  or  creatinine  or  albuminuria  or  microalbuminuria  or  macroalbuminuria  or  proteinuria  or  "kidney  injury")

Google Scholar

("chronic kidney disease" or CKD or "kidney function" or "kidney failure" or "renal disease" or "renal insufficiency" or "renal failure" or "glomerular filtration rate" or "estimated glomerular filtration rate" or creatinine or albuminuria or microalbuminuria or macroalbuminuria or proteinuria or "kidney injury") and ("White matter hyperintensity" or "White matter lesions" or "white matter disease" or "cerebral small vessel disease" or Leukoencephalopathy or "Leuko-araiosis" or "Demyelination of white matter")

Search: **(((((((((((((((chronic kidney disease) OR (CKD)) OR (kidney function)) OR (kidney failure)) OR (renal disease)) OR (renal insufficiency)) OR (renal failure)) OR (glomerular filtration rate (GFR))) OR (estimated glomerular filtration rate(eGFR))) OR (creatinine)) OR (albuminuria)) OR (microalbuminuria)) OR (macroalbuminuria)) OR (proteinuria)) OR (kidney injury)) AND (((((((White matter hyperintensity) OR (White matter lesions)) OR (white matter disease)) OR (cerebral small vessel disease)) OR (Leukoencephalopathy)) OR (Leuko-araiosis)) OR (Demyelination of white matter))**

("renal insufficiency, chronic"[MeSH Terms] OR ("renal"[All Fields] AND "insufficiency"[All Fields] AND "chronic"[All Fields]) OR "chronic renal insufficiency"[All Fields] OR ("chronic"[All Fields] AND "kidney"[All Fields] AND "disease"[All Fields]) OR "chronic kidney disease"[All Fields] OR "CKD"[All Fields] OR (("kidney"[MeSH Terms] OR "kidney"[All Fields] OR "kidneys"[All Fields] OR "kidney s"[All Fields]) AND ("functional"[All Fields] OR "functional s"[All Fields] OR "functionalities"[All Fields] OR "functionality"[All Fields] OR "functionalization"[All Fields] OR "functionalizations"[All Fields] OR "functionalize"[All Fields] OR "functionalized"[All Fields] OR "functionalizes"[All Fields] OR "functionalizing"[All Fields] OR "functionally"[All Fields] OR "functionals"[All Fields] OR "functioned"[All Fields] OR "functioning"[All Fields] OR "functionings"[All Fields] OR "functions"[All Fields] OR "physiology"[MeSH Subheading] OR "physiology"[All Fields] OR "function"[All Fields] OR "physiology"[MeSH Terms])) OR ("renal insufficiency"[MeSH Terms] OR ("renal"[All Fields] AND "insufficiency"[All Fields]) OR "renal insufficiency"[All Fields] OR ("kidney"[All Fields] AND "failure"[All Fields]) OR "kidney failure"[All Fields]) OR ("kidney diseases"[MeSH Terms] OR ("kidney"[All Fields] AND "diseases"[All Fields]) OR "kidney diseases"[All Fields] OR ("renal"[All Fields] AND "disease"[All Fields]) OR "renal disease"[All Fields]) OR ("renal insufficiency"[MeSH Terms] OR ("renal"[All Fields] AND "insufficiency"[All Fields]) OR "renal insufficiency"[All Fields]) OR ("renal insufficiency"[MeSH Terms] OR ("renal"[All Fields] AND "insufficiency"[All Fields]) OR "renal insufficiency"[All Fields] OR ("renal"[All Fields] AND "failure"[All Fields]) OR "renal failure"[All Fields]) OR (("glomerular filtration rate"[MeSH Terms] OR ("glomerular"[All Fields] AND "filtration"[All Fields] AND "rate"[All Fields]) OR "glomerular filtration rate"[All Fields]) AND "GFR"[All Fields]) OR ((("estimability"[All Fields] OR "estimable"[All Fields] OR "estimate"[All Fields] OR "estimated"[All Fields] OR "estimates"[All Fields] OR "estimating"[All Fields] OR "estimation"[All Fields] OR "estimations"[All Fields] OR "estimator"[All Fields] OR "estimator s"[All Fields] OR "estimators"[All Fields]) AND ("glomerular filtration rate"[MeSH Terms] OR ("glomerular"[All Fields] AND "filtration"[All Fields] AND "rate"[All Fields]) OR "glomerular filtration rate"[All Fields])) AND ("erbb receptors"[MeSH Terms] OR ("erbb"[All Fields] AND "receptors"[All Fields]) OR "erbb receptors"[All Fields] OR "egfr"[All Fields])) OR ("creatinin"[All Fields] OR "creatinine"[MeSH Terms] OR "creatinine"[All Fields] OR "creatinines"[All Fields]) OR ("albuminuria"[MeSH Terms] OR "albuminuria"[All Fields]) OR "microalbuminuria"[All Fields] OR "macroalbuminuria"[All Fields] OR ("proteinuria"[MeSH Terms] OR "proteinuria"[All Fields] OR "proteinurias"[All Fields]) OR (("kidney"[MeSH Terms] OR "kidney"[All Fields] OR "kidneys"[All Fields] OR "kidney s"[All Fields]) AND ("injurie"[All Fields] OR "injuried"[All Fields] OR "injuries"[MeSH Subheading] OR "injuries"[All Fields] OR "wounds and injuries"[MeSH Terms] OR ("wounds"[All Fields] AND "injuries"[All Fields]) OR "wounds and injuries"[All Fields] OR "injurious"[All Fields] OR "injury s"[All Fields] OR "injuryed"[All Fields] OR "injurys"[All Fields] OR "injury"[All Fields]))) AND ((("white matter"[MeSH Terms] OR ("white"[All Fields] AND "matter"[All Fields]) OR "white matter"[All Fields]) AND ("hyperintense"[All Fields] OR "hyperintensities"[All Fields] OR "hyperintensity"[All Fields] OR "hyperintensive"[All Fields])) OR (("white matter"[MeSH Terms] OR ("white"[All Fields] AND "matter"[All Fields]) OR "white matter"[All Fields]) AND ("lesion"[All Fields] OR "lesion s"[All Fields] OR "lesional"[All Fields] OR "lesions"[All Fields])) OR ("leukoencephalopathies"[MeSH Terms] OR "leukoencephalopathies"[All Fields] OR ("white"[All Fields] AND "matter"[All Fields] AND "disease"[All Fields]) OR "white matter disease"[All Fields]) OR ("cerebral small vessel diseases"[MeSH Terms] OR ("cerebral"[All Fields] AND "small"[All Fields] AND "vessel"[All Fields] AND "diseases"[All Fields]) OR "cerebral small vessel diseases"[All Fields] OR ("cerebral"[All Fields] AND "small"[All Fields] AND "vessel"[All Fields] AND "disease"[All Fields]) OR "cerebral small vessel disease"[All Fields]) OR ("leucoencephalopathies"[All Fields] OR "leukoencephalopathies"[MeSH Terms] OR "leukoencephalopathies"[All Fields] OR "leucoencephalopathy"[All Fields] OR "leukoencephalopathy"[All Fields]) OR "Leuko-araiosis"[All Fields] OR (("demyelinate"[All Fields] OR "demyelinated"[All Fields] OR "demyelinates"[All Fields] OR "demyelinating diseases"[MeSH Terms] OR ("demyelinating"[All Fields] AND "diseases"[All Fields]) OR "demyelinating diseases"[All Fields] OR "demyelination"[All Fields] OR "demyelinations"[All Fields] OR "demyelinating"[All Fields] OR "demyelinative"[All Fields] OR "demyelinization"[All Fields] OR "demyelinizing"[All Fields]) AND ("white matter"[MeSH Terms] OR ("white"[All Fields] AND "matter"[All Fields]) OR "white matter"[All Fields])))

**Translations**

**chronic kidney disease:** "renal insufficiency, chronic"[MeSH Terms] OR ("renal"[All Fields] AND "insufficiency"[All Fields] AND "chronic"[All Fields]) OR "chronic renal insufficiency"[All Fields] OR ("chronic"[All Fields] AND "kidney"[All Fields] AND "disease"[All Fields]) OR "chronic kidney disease"[All Fields]

**kidney:** "kidney"[MeSH Terms] OR "kidney"[All Fields] OR "kidneys"[All Fields] OR "kidney's"[All Fields]

**function:** "functional"[All Fields] OR "functional's"[All Fields] OR "functionalities"[All Fields] OR "functionality"[All Fields] OR "functionalization"[All Fields] OR "functionalizations"[All Fields] OR "functionalize"[All Fields] OR "functionalized"[All Fields] OR "functionalizes"[All Fields] OR "functionalizing"[All Fields] OR "functionally"[All Fields] OR "functionals"[All Fields] OR "functioned"[All Fields] OR "functioning"[All Fields] OR "functionings"[All Fields] OR "functions"[All Fields] OR "physiology"[Subheading] OR "physiology"[All Fields] OR "function"[All Fields] OR "physiology"[MeSH Terms]

**kidney failure:** "renal insufficiency"[MeSH Terms] OR ("renal"[All Fields] AND "insufficiency"[All Fields]) OR "renal insufficiency"[All Fields] OR ("kidney"[All Fields] AND "failure"[All Fields]) OR "kidney failure"[All Fields]

**renal disease:** "kidney diseases"[MeSH Terms] OR ("kidney"[All Fields] AND "diseases"[All Fields]) OR "kidney diseases"[All Fields] OR ("renal"[All Fields] AND "disease"[All Fields]) OR "renal disease"[All Fields]

**renal insufficiency:** "renal insufficiency"[MeSH Terms] OR ("renal"[All Fields] AND "insufficiency"[All Fields]) OR "renal insufficiency"[All Fields]

**renal failure:** "renal insufficiency"[MeSH Terms] OR ("renal"[All Fields] AND "insufficiency"[All Fields]) OR "renal insufficiency"[All Fields] OR ("renal"[All Fields] AND "failure"[All Fields]) OR "renal failure"[All Fields]

**glomerular filtration rate:** "glomerular filtration rate"[MeSH Terms] OR ("glomerular"[All Fields] AND "filtration"[All Fields] AND "rate"[All Fields]) OR "glomerular filtration rate"[All Fields]

**estimated:** "estimability"[All Fields] OR "estimable"[All Fields] OR "estimate"[All Fields] OR "estimated"[All Fields] OR "estimates"[All Fields] OR "estimating"[All Fields] OR "estimation"[All Fields] OR "estimations"[All Fields] OR "estimator"[All Fields] OR "estimator's"[All Fields] OR "estimators"[All Fields]

**glomerular filtration rate:** "glomerular filtration rate"[MeSH Terms] OR ("glomerular"[All Fields] AND "filtration"[All Fields] AND "rate"[All Fields]) OR "glomerular filtration rate"[All Fields]

**eGFR:** "erbb receptors"[MeSH Terms] OR ("erbb"[All Fields] AND "receptors"[All Fields]) OR "erbb receptors"[All Fields] OR "egfr"[All Fields]

**creatinine:** "creatinin"[All Fields] OR "creatinine"[MeSH Terms] OR "creatinine"[All Fields] OR "creatinines"[All Fields]

**albuminuria:** "albuminuria"[MeSH Terms] OR "albuminuria"[All Fields]

**proteinuria:** "proteinuria"[MeSH Terms] OR "proteinuria"[All Fields] OR "proteinurias"[All Fields]

**kidney:** "kidney"[MeSH Terms] OR "kidney"[All Fields] OR "kidneys"[All Fields] OR "kidney's"[All Fields]

**injury:** "injurie"[All Fields] OR "injuried"[All Fields] OR "injuries"[Subheading] OR "injuries"[All Fields] OR "wounds and injuries"[MeSH Terms] OR ("wounds"[All Fields] AND "injuries"[All Fields]) OR "wounds and injuries"[All Fields] OR "injurious"[All Fields] OR "injury's"[All Fields] OR "injuryed"[All Fields] OR "injurys"[All Fields] OR "injury"[All Fields]

**White matter:** "white matter"[MeSH Terms] OR ("white"[All Fields] AND "matter"[All Fields]) OR "white matter"[All Fields]

**hyperintensity:** "hyperintense"[All Fields] OR "hyperintensities"[All Fields] OR "hyperintensity"[All Fields] OR "hyperintensive"[All Fields]

**White matter:** "white matter"[MeSH Terms] OR ("white"[All Fields] AND "matter"[All Fields]) OR "white matter"[All Fields]

**lesions:** "lesion"[All Fields] OR "lesion's"[All Fields] OR "lesional"[All Fields] OR "lesions"[All Fields]

**white matter disease:** "leukoencephalopathies"[MeSH Terms] OR "leukoencephalopathies"[All Fields] OR ("white"[All Fields] AND "matter"[All Fields] AND "disease"[All Fields]) OR "white matter disease"[All Fields]

**cerebral small vessel disease:** "cerebral small vessel diseases"[MeSH Terms] OR ("cerebral"[All Fields] AND "small"[All Fields] AND "vessel"[All Fields] AND "diseases"[All Fields]) OR "cerebral small vessel diseases"[All Fields] OR ("cerebral"[All Fields] AND "small"[All Fields] AND "vessel"[All Fields] AND "disease"[All Fields]) OR "cerebral small vessel disease"[All Fields]

**Leukoencephalopathy:** "leucoencephalopathies"[All Fields] OR "leukoencephalopathies"[MeSH Terms] OR "leukoencephalopathies"[All Fields] OR "leucoencephalopathy"[All Fields] OR "leukoencephalopathy"[All Fields]

**Demyelination:** "demyelinate"[All Fields] OR "demyelinated"[All Fields] OR "demyelinates"[All Fields] OR "demyelinating diseases"[MeSH Terms] OR ("demyelinating"[All Fields] AND "diseases"[All Fields]) OR "demyelinating diseases"[All Fields] OR "demyelination"[All Fields] OR "demyelinations"[All Fields] OR "demyelinating"[All Fields] OR "demyelinative"[All Fields] OR "demyelinization"[All Fields] OR "demyelinizing"[All Fields]

**white matter:** "white matter"[MeSH Terms] OR ("white"[All Fields] AND "matter"[All Fields]) OR "white matter"[All Fields]
